# Investigation and analysis of pertussis antibody levels among healthy people in Hangzhou, Zhejiang Province from 2021 to 2024

**DOI:** 10.3389/fpubh.2026.1780333

**Published:** 2026-03-13

**Authors:** Yaning Zhuo, Xinren Che, Yuyang Xu, Xuechao Zhang, Yingying Yang, Xiaoping Zhang, Yan Liu

**Affiliations:** 1 School of Public Health, Zhejiang Chinese Medical University, Hangzhou, China; 2 Department of Immunization Program, Hangzhou Center for Disease Control and Prevention (Hangzhou Health Supervision Institution), Hangzhou, Zhejiang, China

**Keywords:** antibody level, healthy people, pertussis, seroepidemiology, vaccine

## Abstract

**Objectives:**

This study aims to investigate the levels of pertussis antibodies among the healthy population in Hangzhou, Zhejiang Province from 2021 to 2024. The goal is to understand the immune status of the population regarding pertussis, providing a basis for assessing the epidemiological risk of pertussis.

**Methods:**

A multi-stage stratified sampling method was employed, categorizing the population into 11 age groups. From 2021 to 2024, one district/county was selected each year to collect blood samples from healthy individuals. Enzyme-linked immunosorbent assay (ELISA) was used to detect serum pertussis Immunoglobulin G (IgG) antibodies.

**Results:**

A total of 1,352 participants were included in the study. A definite history of vaccination was documented in 65.09% of the study subjects. The overall positive rate of antibodies in the healthy population was 37.50%, with a geometric mean concentration (GMC) of 51.54 U/mL (95% CI: 47.82–55.34). Statistically significant differences were observed in the positive rates and GMC values of pertussis IgG across different years, age groups, vaccination histories, and intervals between primary and last doses. Notably, the positive rate and GMC value in 2023 and when the interval between the first and last doses was less than 1 year were the highest within the corresponding subgroups (*p* < 0.05); The GMC values for the 1-year and 2-year age groups were 87.60 IU/mL and 99.01 IU/mL, respectively, which were significantly higher than those in individuals under 1 year and over 10 years of age. No statistically significant differences were found in the positive rates and GMC values of pertussis IgG between different genders and regions (*p* > 0.05).

**Conclusion:**

Suboptimal pertussis antibody levels in specific age groups, particularly among adolescents and adults, may contribute to an increased risk of transmission. Therefore, it may be worthwhile to consider booster vaccinations for these at-risk populations including the introduction of the vaccine for adolescents and adults, such as Tdap vaccine. In parallel, establishing a standardized testing and surveillance system would be essential for evaluating population immunity and mitigating future outbreak risks.

## 1 Introduction

Pertussis, an acute respiratory infectious disease caused by *Bordetella pertussis* (Bp), is characterized by paroxysmal and spasmodic coughing, often accompanied by a characteristic “whooping” sound during inhalation. In some cases, it can progress to severe complications or death. Pertussis is classified as a category B infectious disease in China and is primarily transmitted through respiratory droplets. The incubation and infectious periods are relatively prolonged, leading to widespread susceptibility in the population, particularly with high secondary attack rates within households. It is most commonly observed in children under 5 years of age, especially in infants of low age. Pertussis remains a significant global public health issue and is one of the leading causes of morbidity and mortality in infants worldwide (1, 2).

The primary method for preventing pertussis is vaccination (3). In China, the diphtheria, tetanus, and whole-cell pertussis combined vaccine (DTwP), containing whole-cell pertussis vaccine (wP), was included in the national immunization program in 1978, leading to a significant reduction in pertussis incidence. In 2007, the diphtheria, tetanus, and acellular pertussis combined vaccine (DTaP), with a proven better tolerance profile, began to be gradually used, completely replacing DTwP by 2012 (4, 5). The reported vaccination coverage for the three doses has consistently remained above 99%. However, since the 1980s, countries with high vaccine coverage, such as the United States, the United Kingdom, and Australia (6), have reported increases in pertussis cases. China also faced this public health issue after 2011. From 2006 to 2010, the annual reported incidence of pertussis in China decreased to 0.2/100,000 (7). Recently, the reported incidence has shown an upward trend, rising to between 0.32 and 2.15/100,000 from 2018 to 2021 (8). The reported incidence rate reached 2.71 per 100,000 in 2022, the highest level since 1989. Among them, 52.67% were children aged 0–4, 40.75% were children aged 5–9, and 7.06% were people aged 10 and older (9). The incidence of pertussis among adolescents and adults in China is severely underestimated due to atypical symptoms (10).

Pertussis toxin (PT) is unique to *Bordetella pertussis* (11), and plays a crucial role during the course of pertussis. As one of the primary immunogenic components of the vaccine, antibodies against pertussis toxin serve as indicators for evaluating immune response and for serological diagnosis. Elevated levels of pertussis IgG in the serum of healthy individuals can be regarded as sensitive indicators of recent pertussis infection, and also can be used as one of the indicators for monitoring the vaccine efficacy in individuals who have received the DPT vaccine. Additionally, serological monitoring can help identify mild and atypical cases, allowing for a better estimation of the overall pertussis infection levels in the population (12).

To understand the antibody levels of pertussis in the healthy population of Hangzhou, this study collected serum samples from the healthy population of Hangzhou from 2021 to 2024, retrospectively analyzed the test results and explored the influencing factors, providing a certain scientific basis for the improvement of pertussis prevention and control strategies.

## 2 Subjects and methods

### 2.1 Research object

From 2021 to 2024, the survey sites were different and randomly selected each year, namely Fuyang District, Tonglu County, Binjiang District, and Gongshu District, totaling four districts or counties. A multi-stage stratified sampling method was used to select the resident healthy population as study subjects, categorizing participants into 11 age groups (<1, 1-, 2-, 3-, 5-, 7-, 10-, 15-, 20-, 30-, ≥40). And select several streets according to age groups for the selection of research subjects. Based on the sample size calculation formula: $n={\left(\frac{Z_{1-a/2}}{d}\right)}^{2}p(1-p)$ (*p* = 33.3%, d = 0.1, *α* = 0.05),and considering a reported positive rate of 33.3% for pertussis IgG antibodies (13), a sample size of 328 was calculated for each district, ensuring that each age group included at least 30 participants. Exclusion criteria: individuals who refused to sign the informed consent form or who did not cooperate with the surveillance process. Basic demographic information, including age, gender, vaccination history related to pertussis, and the time interval since the last vaccination, was collected from the Zhejiang Province Immunization Information System. Blood samples were collected from the study subjects to detect pertussis IgG antibodies.

### 2.2 Research methods

All included participants were informed about the study’s purpose and completed a survey questionnaire. Subsequently, 2 mL of peripheral venous blood was drawn, and the samples were centrifuged at 3,000 rpm for 5 min to separate the serum. The isolated serum was stored in cryogenic tubes at −80 °C for long-term preservation. Antibody titers were measured using the WHO-recommended ELISA method. For the period from 2021 to 2024, the reagent kit batch number was EM0253. A concentration of pertussis IgG antibodies below 40 U/mL was considered negative, between 40 and 50 U/mL was classified as a critical value, and above 50 U/mL was deemed positive. Due to the fact that the critical antibody concentration is commonly interpreted as an indicator of waning immunity, which may not provide sufficient protective effects (14), this study assigns the critical value to the negative group for pertussis antibodies. This aims to more directly observe the susceptibility levels of the population to the disease. The definition range of positive and negative results for pertussis IgG antibody were defined according to the instructions of the test kit. And the positive means past pertussis infection, successful vaccination or immunoglobulin recipient. All kits used were sourced from Virion/Serion GmbH, Germany.

### 2.3 Statistical analysis

The data were organized using Microsoft Excel 2020 software, and the overall and subgroup positive rates of pertussis IgG antibodies and GMC were calculated using SPSS version 27. For univariate analysis, the *χ*^2^ test (or Fisher’s exact probability method) and *t*-test (or analysis of variance) were employed. For inter-group comparisons, multiple comparison tests were conducted. If the data followed a normal distribution, ANOVA was selected, followed by Tukey’s test for *post-hoc* comparisons; otherwise, the Kruskal–Wallis test was used, with Bonferroni correction applied. Multivariate analyses were conducted using logistic regression and generalized linear model analysis. A *p*-value < 0.05 was considered statistically significant.

## 3 Ethical research

This study has obtained ethical approval from the Medical Ethics Committee of the Hangzhou Center for Disease Control and Prevention (Hangzhou Institute of Health Inspection) (Approval Number: 2021-18). Under the technical guidance of the local Center for Disease Control and Prevention and health institutions, the notification and sample collection processes in each district or county were ensured. All target participants (parents) were fully informed about the study content and signed informed consent forms before the study commenced.

## 4 Results

### 4.1 Overall pertussis antibody levels

A total of 1,352 participants were included in the study, with males and females comprising 48.37% (654/1,352) and 51.63% (698/1,352), respectively. The urban and rural populations represented 49.19% (665/1,352) and 50.81% (687/1,352). The overall positive rate of pertussis antibodies in the healthy population was 37.50%, with the GMC of 51.54 U/mL (95% CI: 47.82–55.34).

### 4.2 Pertussis antibody levels among different year groups

Statistically significant differences in the positive rates and GMC values of pertussis IgG were observed among different years (*χ*^2^ = 71.03, *H* = 106.65, *p* < 0.01). The antibody positive rate exhibited a trend of first increasing and then decreasing over the years, with the highest positive rate recorded in 2023 at 53.43%. According to multiple comparison results, the pertussis GMC in 2023 was significantly higher than in other years (*p* < 0.05), measuring 72.06 U/mL (95% CI: 65.87–86.25) (see Tables 1, 2).

**Table 1:** The analysis results of the positive rate of pertussis antibodies in each subgroup.

Subgroup	Total (*n*)	Positive case (*n*)	Positive rate (%)	*χ* ^2^	*p*
Year					
2021	357	131	36.69	71.03	<0.01
2022	330	125	37.88		
2023	335	179	53.43		
2024	330	72	21.82		
Gender					
Male	654	259	39.6	2.39	0.12
Female	698	248	35.53		
Age (y)					
<1	122	51	41.8	88.89	<0.01
1~	123	62	50.41		
2~	124	76	61.29		
3~	122	55	45.08		
5~	120	52	43.33		
7~	122	44	36.07		
10~	135	40	29.63		
15~	120	27	22.5		
20~	120	26	21.67		
30~	124	25	20.16		
≥40	120	49	40.83		
District					
Urban	665	251	37.74	0.03	0.86
Rural	687	256	37.26		
Immunization history					
Unvaccinated	37	6	16.22	49.1	<0.01
1 dose	10	1	10		
2 dose	8	1	12.5		
3 dose	505	210	41.58		
4 dose	357	169	47.34		
Unknown	435	120	27.59		
Intervals between primary and last doses (y)					
<1	165	79	47.88	13.15	<0.01
1~	681	294	43.17		
2~	16	5	31.25		
≥3	8	2	25		
Unvaccinated	37	6	16.22		

**Table 2:** The analysis results of the GMC of pertussis antibodies in each subgroup.

Subgroup	GMC [U/ml, (95% CI)]	*Z*/*H*	*p*
Year			
2021	47.54 (40.99–54.09)	*H* = 106.65	<0.01
2022	50.02 (43.74–56.30)		
2023	72.06 (65.87–86.25)		
2024	32.49 (26.83–38.15)		
Gender			
Male	54.22 (48.48–59.96)	*H* = 0.60	0.44
Female	48.02 (44.08–53.97)		
Age (y)			
<1	51.13 (39.99–62.26)	*H* = 85.27	<0.01
1~	87.60 (66.75–108.45)		
2~	99.01 (77.94–118.08)		
3~	61.76 (49.20–74.32)		
5~	47.38 (37.77–56.99)		
7~	46.42 (36.64–56.21)		
10~	41.72 (30.47–52.96)		
15~	29.74 (24.14–35.34)		
20~	30.55 (24.31–36.79)		
30~	26.62 (22.31–30.94)		
≥40	45.51 (38.23–52.79)		
District			
Urban	54.44 (48.37–60.50)	*H* = 0.30	0.58
Rural	48.73 (44.19–53.27)		
Immunization history			
Unvaccinated	20.93 (11.59–30.27)	*H* = 61.04	<0.01
1 dose	15.67 (4.87–26.45)		
2 dose	14.93 (3.72–26.13)		
3 dose	62.56 (54.57–70.56)		
4 dose	61.92 (54.74–69.10)		
Unknown	37.32 (31.05–37.59)		
Intervals between primary and last doses (y)			
<1	63.27 (52.40–74.15)	*H* = 22.64	<0.01
1~	62.51 (56.01–68.90)		
2~	34.38 (20.66–48.10)		
≥3	32.57 (−0.06–65.19)		
Unvaccinated	20.93 (11.59–30.27)		

### 4.3 Pertussis antibody levels in different gender groups

The positive rate of pertussis IgG in males was 39.6%, while in females it was 35.53%, with no statistically significant difference observed (*χ*^2^ = 2.39, *p* = 0.12). The GMC was 54.22 IU/mL (95% CI: 48.48–59.96) for males and 48.02 U/mL (95% CI: 44.08–53.97) for females, also showing no statistically significant difference (*H* = 0.60, *p* = 0.44) (see Tables 1, 2).

### 4.4 Pertussis antibody levels among different age groups

Statistically significant differences in the positive rates and GMC values of pertussis IgG were observed among different age groups (*χ*^2^ = 88.89, *H* = 85.27, *p* < 0.01). With the increase of age, the positive rates and GMC exhibited a trend of initially increasing, then decreasing, and subsequently increasing again. The top three positive rates were the 2-year-old group, the 1-year-old group, and the 3-year-old group, which were 61.29, 50.41, and 45.08% respectively; The top three GMC values were the 2-year-old group, the 1-year-old group and the 3-year-old group, which were 99.01 U/mL (95% CI:77.94–118.08), 87.60 U/mL (95% CI:66.75–108.45) and 61.76 U/mL (95% CI:49.20–74.32) respectively. According to multiple comparison results, the GMC for the 1-year-old group was significantly higher than that of the group <1-year-old group and the ≥5-year-old group, while the GMC for the 2-year-old group was significantly higher than all other age groups except for the 1-year-old group (*p* < 0.05) (see Tables 1, 2).

### 4.5 Pertussis antibody levels in different district groups

The positive rate of pertussis IgG in urban areas was 37.74%, while in rural areas it was 37.26%, with no statistically significant difference observed (*χ*^2^ = 0.03, *p* = 0.86). The GMC was 54.44 U/mL (95% CI:48.37–60.50) for urban areas and 48.73 U/mL (95% CI:44.19–53.27) for suburban areas, also showing no statistically significant difference (*H* = 0.30, *p* = 0.58) (see Tables 1, 2).

### 4.6 Pertussis antibody levels among different immunization history groups

Statistically significant differences in the positive rates and GMC values of pertussis IgG were observed among the different vaccination history groups (*χ*^2^ = 49.10, *H* = 61.04, *p* < 0.01). A total of 65.09% (880/1,352) of the participants had a definite history of vaccination, 2.74% (37/1,352) had no history of vaccination, and for 32.17% (435/1,352), the vaccination history was undetermined. The highest antibody positive rate was found in the population who received four doses of pertussis-related vaccine, and the lowest was in those who received only one dose, which were 47.34% and 10.00% respectively; The highest GMC value was in the population with three doses, and the lowest was in the population with two doses, which were 62.56 U/mL (95% CI:54.57–70.56) and 14.93 U/mL (95% CI:3.72–26.13) respectively. According to multiple comparison results, the GMC values for individuals receiving three and four doses were significantly higher than those for unvaccinated individuals or those with unclear vaccination history (*p* < 0.05) (see Tables 1, 2).

### 4.7 Pertussis antibody levels among different intervals between primary and last doses groups

Statistically significant differences in the positive rates and GMC values of pertussis IgG were observed among the intervals between the first and last doses of vaccination (*χ*^2^ = 13.15, *H* = 22.64, *p* < 0.01). The positive rate and GMC value showed a downward trend with the increase of the interval time. The positive rate and GMC value of pertussis vaccination with an interval of less than 1 year were the highest, which were 47.88% 和 63.27 U/mL (95% CI:52.40–74.15) respectively (see Tables 1, 2).

### 4.8 Multiple-factor analysis

The results of logistic analysis indicated that the following factors significantly influenced the positive rate of pertussis IgG in the healthy population:1-year-old group, 2-year-old group, ≥20-year-old group, having an immunization history, and the time interval between the first and last doses of the vaccine < 1 year group and 1-year-group; Generalized linear model analysis revealed that factors affecting the GMC of pertussis IgG antibodies included district, age, 3-dose vaccination, 4-dose vaccination and unknown immunization history, as well as the group with the time interval between the first and last doses < 1 year and the 1-year- group (see Table 3).

**Table 3:** Multiple-factor analysis of pertussis IgG antibody levels in healthy populations in Hangzhou from 2021 to 2024.

Subgroup	Positive rate			GMC		
	OR	95% CI	*p*	OR	95% CI	*p*
Gender (Reference: male)						
Female	1.02	0.81–1.30	0.86	1.06	0.95–1.17	0.32
District (Reference: urban)						
Rural	1.11	0.88–1.41	0.37	0.86	0.77–0.95	<0.05
Age (Reference: <1)						
1~	1.98	1.09–3.27	0.1	1.33	1.02–1.72	<0.01
2~	1.61	0.73–3.56	0.24	1.46	1.13–1.89	<0.01
3~	2.28	1.02–5.07	0.04	0.78	0.59–1.03	<0.01
5~	0.98	0.44–2.19	0.96	0.66	0.51–1.86	<0.01
7~	0.89	0.40–2.00	0.79	0.61	0.47–0.80	<0.01
10~	0.66	0.29–1.47	0.31	0.53	0.40–0.70	<0.01
15~	0.4	0.18–0.90	0.03	0.41	0.30–0.57	<0.01
20~	0.35	0.19–0.66	<0.01	0.45	0.31–0.64	<0.01
30~	0.8	0.22–0.68	<0.01	0.39	0.28–0.57	<0.01
≥40	0.35	0.20–0.63	<0.01	0.71	0.50–1.02	<0.01
Immunization history (Reference: unvaccinated)						
1dose	0.2	0.07–0.56	<0.01	0.61	0.30–1.12	0.17
2dose	0.08	0.01–0.68	0.02	0.57	0.27–1.22	0.45
3dose	0.1	0.01–0.89	0.04	2.4	1.65–3.48	<0.01
4dose	0.76	0.39–1.47	0.41	3.25	2.22–4.78	<0.01
Unknown	1.7	0.89–3.24	0.11	2.62	1.84–3.71	<0.01
Intervals between primary and last doses (Reference: unvaccinated)						
<1	4.35	1.73–10.95	<0.01	2.90	1.98–4.24	<0.01
1~	3.93	1.62–9.53	<0.01	2.99	2.09–4.26	<0.01
2~	2.35	0.60–9.26	0.23	1.64	0.87–3.09	0.12
≥3	1.72	0.28–10.68	0.56	1.56	0.68–3.54	0.29

## 5 Discussion

The phenomenon whereby pertussis, previously characterized by low incidence or minimal fluctuations, suddenly exhibits a sustained and significant increase in cases—even escalating to outbreaks—is referred to as the “reemergence of pertussis” (15). Since 2015, the annual reported cases and incidence rate of pertussis in China have shown a continuous upward trend, peaking in 2019. Although the number of pertussis cases declined after 2019, an upward trend was observed again starting in 2022. It is reported that from January to February 2024, the number of pertussis cases in China has reached 32,380, approaching the total number of cases reported in 2022. These data indicate that pertussis has once again become a public health concern (16). Therefore, this study selected the period from 2021 to 2024, during which serum pertussis antibodies were tested in 1,352 healthy individuals from four districts (counties) of Hangzhou. The aim was to assess the population-level pertussis immunity in the context of pertussis reemergence and to analyze associated influencing factors. The results showed that the overall pertussis seropositivity rate from 2021 to 2024 was 37.50%, which did not reach the national protective threshold of 75% based on pertussis IgG levels (17). This result is consistent with findings from other regions, such as those reported in studies conducted in Shanghai and Shijiazhuang (18, 19). The overall GMC was 51.54 U/mL (95% CI:47.82–55.34). In 2023, the levels of pertussis antibodies were the highest, with a positive rate and GMC of 53.43% and 72.06 U/mL (95% CI: 65.87–86.25), respectively. A systematic review indicates that COVID-19 is likely to have contributed to the decline in antibodies against specific pathogens. This decline is attributed to the significant reduction in population exposure to the external environment during the COVID-19 pandemic, which interrupted natural infections and led to decreased antibody levels (20). Additionally, during this period, routine vaccination rates for both children and adults significantly decreased, creating potential immunity gaps that could result in increased pertussis incidence (21). These two factors may, to some extent, explain the elevated levels of pertussis antibodies observed in 2023. There were no statistically significant differences in the positivity rates or GMC among different districts, suggesting a high coverage rate of pertussis vaccination.

There were statistically significant differences in pertussis antibody seropositivity rates and GMC values among different age groups. The results of this survey indicate that overall pertussis antibody levels peak in the 2-year-old group, followed by a declining trend with increasing age. According to the 2021 edition of the National Immunization Program for Children, three types of vaccines containing pertussis components are currently used in China: DTaP, DTaP-Hib, and DTaP-IPV/Hib. All are administered as a four-dose schedule, with primary immunization at 3, 4, and 5 months of age, and a booster dose at 18 months (22). The studies have shown that antibody levels peak within 1 year after children receive four doses of DTaP-containing vaccine, but decline significantly over the subsequent 1–2 years (23, 24). Moreover, a prospective study conducted abroad involving over 1,000 children aged 0–7 years over a decade period showed that the protective efficacy decreased to approximately 50% by the ages of 4 and 5 years (25). This finding is consistent with the results of the present survey, suggesting that the increase in antibody levels observed in the 1–2 year-age-group is primarily attributable to vaccine-induced immune responses. Multiple-factor analysis indicated that age is one of the key factors influencing pertussis antibody levels, with antibody levels decreasing as age increases. This finding is consistent with the conclusion of Wu et al. (26), which suggested that the duration of protection provided by the acellular pertussis vaccine is relatively limited, and its immunological effectiveness diminishes over time. These results suggest that the current vaccination strategy may not be sufficient to curb the spread of pertussis. Both domestic and overseas studies have reported that the actual infection and morbidity rates of pertussis among adolescents and adults are underestimated (27), and that pertussis antibody levels may decline to 0%–20% of the original level within decade (28). To address immunity in these populations, foreign experts have proposed a lifelong immunization strategy. On the basis of completing the original immunity program, an additional dose should be administered around the ages of 5, 10, and 20 respectively, and then every 10 years thereafter, in order to enhance the antibody levels of adolescents and adults (29). Furthermore, given that pertussis antibody levels among Chinese adults are low and maternal antibodies in newborns may be insufficient to protect against infection, some studies have recommended that pregnant women receive a booster dose during each pregnancy, either in the second or third trimester (30). This immunization strategy can help infants who have not obtained sufficient maternal protective antibodies to reduce the risk of pertussis.

Statistically significant differences were observed in pertussis antibody seropositivity rates and the GMC among individuals with different immunization histories. Receiving three or more doses of pertussis-containing vaccines was associated with higher antibody levels, and those who completed the full course of immunization exhibited higher antibody levels compared to those with only partial immunization. Previous studies have demonstrated that the overall protective efficacy of pertussis vaccines can reach 90.75% (31), making vaccination a major determinant of pertussis outcomes. The reemergence of pertussis is closely linked to the three key elements of infectious disease epidemiology: source of infection, transmission route, and susceptible population. While the increase in sources of infection and changes in transmission routes are difficult to control, a study of close contacts of pertussis cases found that 8%–83% of respiratory samples carried BP (32, 33), indicating that reducing the susceptible population is one of the most feasible and effective interventions. Completing the full immunization schedule reduces the number of susceptible individuals, promotes herd immunity, and interrupts transmission chains, thereby decreasing the number of sources of infection. The level of pertussis in patients with an unknown immunization history was relatively high in this research results. This might be due to the fact that most of the people in this study were older and had likely acquired natural infection during periods of pertussis circulation, which was similar to the results of related studies at home and abroad (34, 35). The protective effect of vaccination is not only related to the number of doses received, but also may be influenced by vaccine type and immunization schedule (36). The results of this study also support this view, indicating that the Intervals between primary and last vaccine doses is one of the factors influencing pertussis antibody levels. Intervals between primary and last. Therefore, to maintain vaccine-induced protection, it is essential to timely complete the full course of pertussis-containing vaccine immunization.

Our study has several limitations. First, the assay kit used in this study is a mixed antigen kit, which has relatively low specificity. While ELISA can measure antibody levels, there is no recognized standard for defining a protective titer for pertussis IgG. Due to its low specificity, this assay may lead to an underestimation of vaccine-induced protective efficacy. Additionally, this study only conducted a combined analysis of different types of pertussis-related vaccines (37, 38) and did not distinguish between the types of pertussis vaccines administered to the study population, which lacked further analysis of the immunological effects of different pertussis vaccines. Finally, as the Zhejiang Province immunization information system has only been in use since 2004, all vaccination histories in this study were obtained from this system. Therefore, the vaccination history for individuals in older age groups could not be verified.

In summary, variations in pertussis antibody levels are associated with district, age, immunization history, and the timing of vaccination. To prevent pertussis and address the issue of its reemergence, optimizing immunization program management in rural areas—where the GMC levels of pertussis antibodies are relatively low—may effectively reduce the risk of transmission between urban and rural populations. Furthermore, while ensuring adequate immunization coverage among children, greater attention should be paid to pertussis prevention in adolescents and adults, and efforts should be made to enhance the uptake of pertussis-related vaccines in these groups, thereby establishing a more effective pertussis prevention and control system. The development of new vaccines with longer duration of protection and higher immunogenicity will be crucial in addressing the issue of waning pertussis antibody levels.

## Data Availability

The original contributions presented in the study are included in the article/supplementary material, further inquiries can be directed to the corresponding author.
