# Correction: Investigation and analysis of pertussis antibody levels among healthy people in Hangzhou, Zhejiang Province from 2021 to 2024

**DOI:** 10.3389/fpubh.2026.1836572

**Published:** 2026-07-08

**Authors:** Yaning Zhuo, Xinren Che, Yuyang Xu, Xuechao Zhang, Yingying Yang, Xiaoping Zhang, Yan Liu

**Affiliations:** 1 School of Public Health, Zhejiang Chinese Medical University, Hangzhou, China; 2 Department of Immunization Program, Hangzhou Center for Disease Control and Prevention (Hangzhou Health Supervision Institution), Hangzhou, Zhejiang, China

**Keywords:** antibody level, healthy people, pertussis, seroepidemiology, vaccine

Authors Xinren Che, Yuyang Xu, Xuechao Zhang, Yingying Yang, Xiaoping Zhang were erroneously assigned as equal contributing authors.

The original version of this article has been updated.

